# Mandarin–Italian Dual-Language Children’s Comprehension of Head-Final and Head-Initial Relative Clauses

**DOI:** 10.3389/fpsyg.2020.01379

**Published:** 2020-08-11

**Authors:** Shenai Hu, Francesca Costa, Maria Teresa Guasti

**Affiliations:** ^1^Department of Foreign Language Education, Xiamen University, Xiamen, China; ^2^Department of Psychology, University of Milano-Bicocca, Milan, Italy

**Keywords:** dual-language development, Mandarin, Italian, relative clause comprehension, head-directionality

## Abstract

The acquisition of languages by children using two languages is a matter of debate as many factors contribute to the success of this type of acquisition. We focus on how the competence of dual-language children changes in their two languages as a function of length of exposure and establish whether there are reciprocal influences during language development. We examined the comprehension of subject and object relative clauses in a group of 6-year-old (younger) and 8-year-old (older) Mandarin–Italian dual-language children. After 3 years of regular and intensive exposure to Italian, the younger group reached the same level of competence in the comprehension of relative clauses in their two languages, and after 5 years of exposure to Italian, the older group had a better comprehension of relative clauses in Italian than in Mandarin. Acquiring two languages leads to bidirectional influence, beyond a reciprocal support. Finally, some penalty may be observed in the acquisition of subject head-final relative clauses, which is not evident in that of subject head-initial relative clauses.

## Introduction

The unique way in which dual-language children^1^ develop is difficult to characterize because many variables contribute to shaping their competence. First, the age of onset of dual-language acquisition impacts on several aspects of late language competence (e.g., Flege et al., 1999; Kovelman et al., 2008; Unsworth, 2013). Second, the input, such as which language is most commonly spoken around the child, may determine language dominance (David and Wei, 2008; Hoff et al., 2012; Unsworth et al., 2014; Unsworth, 2016). Third, the characteristics of the surrounding community speaking the child’s L1 may also determine aspects of language development (He, 2006; Montrul, 2012). Finally, cross-linguistic influences have often been observed in dual-language children (Müller and Hulk, 2001; Serratrice et al., 2004), with different transfer effects depending on the types of languages involved (Blom et al., 2012; Zdorenko and Paradis, 2012). In the light of this complicated array of factors at play in the development of dual-language children, we would like to focus on how the competence of these children changes as the length of exposure to the majority language increases. In addition, as in other studies, we intend to establish whether there are reciprocal influences between two typologically distant languages and how these manifest. To achieve these goals, we investigated the comprehension of a complex structure: relative clauses (RCs) in Mandarin–Italian dual-language children. The children were born in Italy; they were first exposed to Mandarin at home and in the Chinese community and started to be regularly exposed to Italian between 2 and 4 years of age in public preschools. At the time of testing, they were all attending an Italian public school and receiving formal instruction in Italian. On weekends (for one or two full days) and during vacation (for 2 months with five full days per week), they attended the community school, where they spoke Mandarin and learned Chinese characters. Two groups of children were involved: the younger group consisted of children attending the last year of preschool or grade 1 in Italian primary school and had a mean age of 6 years; the older group, comprised of children attending grade 2 or 3 in Italian primary school, had a mean age of 8 years. Thus, the first group had less exposure to Italian than the second and was less literate in both languages.

The article is organized as follows. First, we describe Mandarin and Italian RCs and provide a brief review of previous studies with monolingual children and dual-language children. Then, we introduce the current study, report the results, and offer a general discussion.

### Typological Differences Between Mandarin and Italian Relative Clauses

Both Mandarin and Italian have the same canonical word order, namely, subject-verb-object (SVO), but their RCs have different word orders. Mandarin RCs are head-final, with the RC linearly preceding the relativizer *de* and the relative head, while Italian RCs are head-initial, with the RC linearly following the relative head and the relativizer *che* (that/who), as in (1–2).


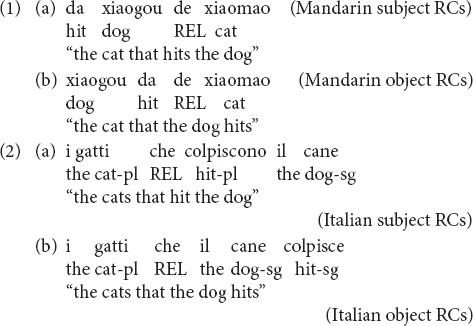


In Mandarin, subject RCs have a verb-object-subject (VOS) order (1a), while object RCs have the SVO order, which corresponds to the canonical order in declarative sentences. In Italian, subject RCs have the SVO order, which is the canonical order in declarative sentences, while object RCs (2b) display the object-subject-verb (OSV) order^2^.

Although the linear order of Mandarin and Italian RCs is different, at the hierarchical level, they present similarities. In (3a) we have the structure of a Mandarin subject RC and in (3b) that of an object RC. In these structures, the relative head, *xiaomao* “cat” must be connected to its copy (indicated with *t*) to be properly interpreted. This dependency in the object RC (3b) crosses the embedded subject *xiaogou* “dog,” namely, the subject structurally intervenes between the relative head and its copy. In contrast, in the subject RC (3a), nothing intervenes between the relative head and its copy. It is also the case that in object RCs, the structural distance between the relative head and its copy is longer than in subject RCs.


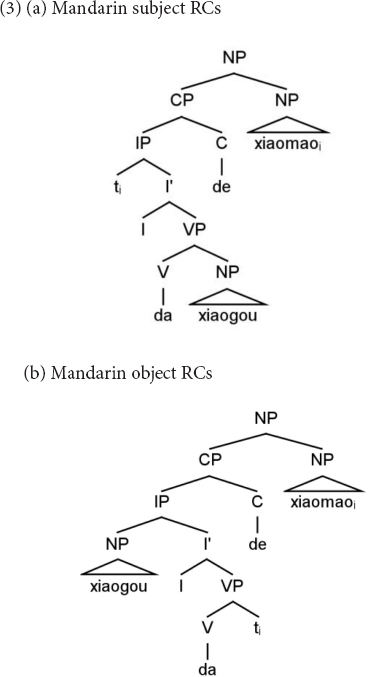


Similarly, in Italian object RCs (4b), the subject intervenes in the dependency between the relative head *i gatti* “the cats” and its copy, while nothing intervenes in the dependency between the relative head and its copy in subject RCs (4a). In addition, the relative head and its copy are hierarchically more distant in object RCs than in subject RCs.


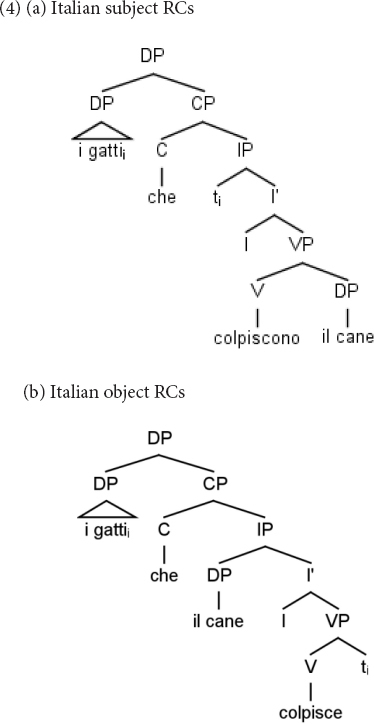


### Comprehension of Relative Clauses by Monolingual Chinese and Italian Children

In this section, we concentrate on the comprehension of RCs in monolingual Mandarin-speaking children (e.g., Lee, 1992; Cao et al., 2005; Hu, 2014; Hu et al., 2016; He et al., 2017; Yang et al., 2020) and monolingual Italian-speaking children (e.g., Arosio et al., 2009, 2011; Adani, 2011; Belletti et al., 2012; Contemori and Belletti, 2014). We will show that in Italian a subject RC advantage is uniformly observed; in Mandarin, the same advantage is observed in most studies, and its absence is likely due to methodological choices.

A subject RC advantage has been reported in the comprehension of Mandarin RCs (e.g., Hu et al., 2016). Hu et al. (2016) tested Mandarin-speaking children aged 3–8, using a character-sentence matching task (i.e., a referent selection task), in which children were asked to point out a character in a set of two pictures including four characters (in one picture character A was acting on character B and in the other, B was acting on A). The results showed that, at least up to 7 years of age, children comprehended subject RCs significantly better than object RCs (e.g., age 7: 99.4% vs. 45.6%), and children at age 8 achieved ceiling performance in both structures. Moreover, the error pattern found in the comprehension of subject RCs differed from that observed in the comprehension of object RCs. In the first case, children aged 3–5 made three different types of errors with equal frequency. In other words, when they failed to choose the correct character, they chose one of the other three characters, which suggests that they were performing randomly. In object RC comprehension, the most common mistake made by children from age 3–7 was the Embedded Error, i.e., children chose the correct picture, but the wrong character. In linguistic terms, this error consists in taking the embedded subject, i.e., the first NP encountered, as the Agent and misinterpreting the sentence (1b), repeated here, *the cat that the dog hits* as *the dog that hits the cat*, that is, they turned an object RC into a subject RC. In some studies, this error is called Head Error (e.g., Kidd et al., 2015; Tsoi et al., 2019). The subject advantage has been replicated in other studies that we will discuss in the next section with monolingual Mandarin-speaking control children (Chan et al., 2017; Tsoi et al., 2019).

However, some other studies on Mandarin RCs report contrasting results regarding the subject advantage, likely due to the types of tasks employed (Cao et al., 2005; Hu, 2014; He et al., 2017). Using a picture-sentence matching task (i.e., a picture selection task), in which children were asked to point to one picture out of two, either an object RC preference or no preference was found (Hu, 2014; He et al., 2017). As pointed out in the literature (Arnon, 2005; Adani, 2011), the use of the picture-sentence matching task is inadequate, as the RC is used to select a character and not a picture. Moreover, this task overestimates children’s comprehension of RCs, especially in head-final ones as in Mandarin (Hu, 2014; Hu et al., 2016). To find the matching picture, children could simply rely on the linear order of the embedded prenominal RC, which is VO for subject RCs and SV for object RCs. In this way, they can choose the correct picture even if they do not choose the correct character. Finally, the picture-sentence matching task does not offer the possibility of observing the error types found in the character-sentence matching task.

In the comprehension of Italian RCs, a subject RC advantage is uniformly reported in children at least up to age 6, regardless of the task used, and afterward ceiling effects of object RCs are reported (Arosio et al., 2009, 2011; Adani, 2011). This preference is found in many studies on the acquisition of head-initial RCs (e.g., Friedmann et al., 2009). Adani (2011) tested Italian-speaking children aged 3–7 with a character-sentence matching task, in which children were asked to point out a character in a picture involving three characters. Italian-speaking children until age 6 comprehended subject RCs more accurately than object RCs (e.g., age 3: 91% vs. 53%). Note that children at age 3 achieved almost ceiling performance in subject RCs, whereas children until age 7 did so in object RCs. If children failed to understand RCs, they mainly made Reversal Error. In the case of object RCs, Reversal Error consists in taking the relative head, i.e., the first NP encountered, as the Agent and misinterpreting the sentence (2b) *the cats that the dog hits* by pointing to the characters described by *the cats that hit the dog*. In this case, the theta-roles are reversed and again an object RC is turned into a subject RC.

One interpretation of the subject advantage is that it results from the fact that the structural distance between the relative head and its copy is shorter in subject RCs (see 3a and 4a) than in object RCs, both in Mandarin and Italian. In addition, in subject RCs nothing intervenes in this dependency, while in object RCs the embedded subject intervenes in this dependency (i.e., it c-commands the object copy) (Friedmann et al., 2009). As subject RCs are less complex than object RCs, it is not surprising that children’s mistakes in both languages consist of turning object RCs into subject RCs. In fact, Reversal Errors in Italian occur because children take the relative head, which comes first in the sentence and in the hierarchical structure, as the Agent of the action and the Subject of the sentence. In Mandarin, Embedded Errors come about because children take the embedded subject as the Agent and Subject: given the order of Mandarin object RCs (S V < O > REL O), the first NP is encountered linearly and hierarchically.

These similarities notwithstanding, comparing the Mandarin and the Italian studies, two differences stand out. First, Italian-speaking children showed ceiling performance in the comprehension of subject RCs as of age 3 (Adani, 2011), while Mandarin-speaking children did so only at age 7 (Hu et al., 2016). Second, while contrasting results are reported for the comprehension of Mandarin RCs, this is not the case in the comprehension of Italian RCs. In our view, this asymmetry is mainly due to the different tasks used, which have an impact when it comes to the comprehension of head-final RCs (as in Mandarin), but not when the comprehension of head-initial RCs is at stake.

### Relative Clauses in Dual-Language Children

The comprehension of RCs by dual-language children has been investigated in a number of studies (e.g., Garraffa et al., 2015; Hopp et al., 2019). Here, we concentrate on the studies focused on language pairs whose RCs have opposite orders, namely, head-initial RCs (such as English and Italian) vs. head-final RCs (such as Mandarin, Cantonese, and Korean) (e.g., Lee and Lee, 2004; Kidd et al., 2015; Chan et al., 2017; Hu and Guasti, 2017; Tsoi et al., 2019; Jia and Paradis, 2020). As we will see, these studies provide an inconsistent picture, either a subject/object asymmetry or no asymmetry being reported in dual-language acquisition; the error patterns of dual-language children are different from that of monolingual children, but it is not clear how this difference is related to length of exposure and thus to transfer effects.

Kidd et al. (2015) investigated the comprehension of RCs in simultaneous Cantonese–English-speaking children (*M* = 8;11, age range: 4;10–11;11) living in Australia, using a character-sentence matching task. The results revealed a subject RC advantage in the dual-language children, but no advantage in monolingual Cantonese-speaking children. The difficulty that the dual-language children experienced with Cantonese object RCs was attributed to the fact that Cantonese object RCs display the canonical SVO word order, which competes with the canonical SVO word order of declarative sentences in Cantonese and, crucially, in English. This competition was responsible for the subject advantage. Typically, the dual-language children made more Embedded Errors than the monolingual children when they were presented with an object RC. This means that when listening to an object RC, the children assigned the Agent thematic role to the first NP in Cantonese and stuck to this interpretation, which is also supported by their English. One weakness of the study is the large age range of the children tested, namely, from age 4 to age 11, which may conceal different patterns across ages and thus length of exposure to the languages. In other words, it is difficult to see how languages influence each other and whether the dual-language effect is consistent across ages (Hu and Guasti, 2017).

Investigating the comprehension of Mandarin and Italian RCs by Mandarin–Italian dual-language children (aged 6;0–9;11) and their age-matched monolingual peers, Hu and Guasti (2017) found a subject RC advantage in both languages, similar to their monolingual peers, but lower accuracy rates than the monolingual children. The authors proposed that learning two languages may slow down the acquisition of complex structures such as RCs. However, they did not provide any independent measure of language competence to discard the conjecture that dual-language children’s competence was generally weaker than that of monolingual children.

Two other relevant studies investigating Mandarin RC comprehension are Tsoi et al. (2019) and Jia and Paradis (2020), both testing Mandarin–English dual-language children, but showing a different comprehension pattern. Jia and Paradis (2020) showed that dual-language school-aged children (*M* = 8;0) in Canada were comparable to monolingual Mandarin-speaking children (*M* = 7;1) in comprehending subject and object RCs. However, their dual-language children were older than their monolingual children. In addition, the authors used a picture-sentence matching task, which, as mentioned earlier, may overestimate children’s abilities in the comprehension of RCs, especially in Mandarin. Tsoi et al. (2019) tested two groups of Mandarin–English dual-language children living in Australia, and compared them with language-matched (receptive vocabulary) monolingual Mandarin-speaking children. For Mandarin, they found a subject RC advantage in all the groups, consistent with the results of the aforementioned studies (Hu et al., 2016; Hu and Guasti, 2017). In English, the younger dual-language children (*M* = 6;1) had more difficulties with RCs than the older ones (*M* = 8;9); in addition, a subject advantage was evident in the younger group, but not in the older one, due to ceiling effects. Regarding the issue of reciprocal influence and based on the fact that monolingual Mandarin-speaking children displayed a subject advantage, Tsoi et al. (2019) concluded that the subject RC advantage in Mandarin-speaking dual-language children cannot be attributed to the effect of dual-language use, contra Kidd et al. (2015). However, Tsoi et al. (2019) noted that influences between the languages were evident in that the error pattern of the dual-language children was different from that of the monolinguals. Embedded Errors were the most common type of error, both in Mandarin and English. In both languages, the younger children made more Embedded Errors than the older ones and they made them more often in object RCs than subject RCs. Embedded Errors are rare in monolingual children acquiring head-initial RCs, but were found when the dual-language children failed to comprehend English RCs. In Mandarin, Reversal Errors were also found more frequently in the dual-language children than the monolinguals, and more in the younger children than the older children. Notice that Reversal Errors are typically found in the monolingual acquisition of head-initial RCs, as we said earlier (Adani, 2011).

Relative clauses comprehension has also been studied in multilingual acquisition contexts. Chan et al. (2017) compared a group of trilingual Cantonese (L1)-English (L2)-Mandarin (L3) children (*M* = 5;8) to monolingual Mandarin-speaking children and monolingual Cantonese-speaking children, and found a subject advantage in Mandarin for the trilingual and the monolingual Mandarin speakers. In Cantonese, again, the trilingual children displayed a subject advantage, but their monolingual Cantonese-speaking peers displayed an object advantage, contrary to the lack of advantage reported in Kidd et al. (2015). Embedded Errors were more frequent than Reversal Errors, both in Mandarin and Cantonese, in line with studies on monolingual Mandarin and Cantonese speakers.

In sum, in the comprehension of RCs in dual-language children with typologically different languages, some contrasts are apparent. First, Tsoi et al.’s (2019) study calls into question the claim that the subject RC advantage found in dual-language children is due to the effect of acquiring a language with head-initial RCs. However, there are contrasting results (Kidd et al., 2015) and few studies have focused on pairs of languages with RCs displaying different orders, and thus, this claim needs further support. Second, the effect of dual-language learning seems to be evident in the types of errors, but to understand better how this influence shapes acquisition, it is important to see how the type of error changes as a response to length of exposure. Third, dual-language children may have a weaker competence than monolingual children in understanding RCs in one or both of their languages, and this may be due to the influence of one language on the other or to the quantity of input. As we observed in the previous section, head-final subject RCs seem to be more difficult than head-initial RCs, in spite of a subject advantage.

### Aims of the Current Study and Predictions

In this study, we aim at (i) investigating the role of the length of exposure to the majority language in shaping the comprehension of RCs, (ii) examining the reciprocal influence of the two typologically distant languages, especially at the level of errors, again as a function of the length of exposure, and (iii) gaining insight into the delay in comprehending subject RCs in Mandarin. To achieve these goals, we recruited two groups of Mandarin–Italian dual-language children, one who had been exposed to the majority language for about 3 years (the younger group) and the other for about 5 years (the older group). While the former had little formal education in Italian, the latter had more. We chose RCs as this structure is a late acquisition even in monolingual children and, therefore, we could be sure that it was still developing in the participating children. In addition, since RCs present an “opposite” word order in the two languages (i.e., head-final in Mandarin and head-initial in Italian), they allow us to investigate the issue of reciprocal influence. The first two aims have been addressed in other studies, but having two groups will better shed light on the role of length of exposure and transfer effects. Besides, given the contrasting results, replication is needed. Finally, we will contribute new data from an L2, Italian, rarely investigated in dual-language acquisition, and focus more on error analysis.

We expect to replicate previous findings concerning the subject RC advantage in both languages, especially in the younger group. Given that 5–7 years of formal education are needed to achieve a literacy level comparable to those of monolingual children (Cummins, 1979) and that RCs are formally taught in Italian primary school, we may anticipate that the older group would not display a subject RC advantage in Italian because of ceiling performance. It is possible that the advantage is still evident in Mandarin. In fact, although the older group of children speak Mandarin at home and are literate in Mandarin, their competence is likely lower than that of monolingual children, as they attend Mandarin classes only on weekends and vacations. We also anticipate that Mandarin RCs will lag behind Italian RCs, as children are more often exposed to Italian, which is the majority language.

The reciprocal influence may be evident in the fact that better comprehension in one language correlates with better comprehension in the other. This influence will also manifest at the level of errors. As we pointed out in the earlier discussion, two main types of errors have been reported in the literature: Reversal Errors, which consist of reversing the thematic roles; and Embedded Errors, which consist of choosing the embedded argument as the relative head. First, we expect the younger group to behave as monolingual Mandarin-speaking children, as far as Mandarin is concerned, as this may still be their dominant language and they had less experience with Italian than the older group. Specifically, for Mandarin subject RCs, we expect the younger group to make an equal number of both types of errors, in line with the previous studies. Both types of errors may also be evident in Italian. Although Italian subject RCs display the canonical order (SVO), this order in Mandarin is that of an object RC and this may lead children to err and choose the referent randomly. Second, in the case of object RCs, both types of errors result in a subject RC. If the subject advantage is the result of subject RCs being structurally less complex, we expect the younger group to adopt the processing strategy of taking the first NP as the Agent, in line with the fact that both Italian and Mandarin are SVO languages. As a result, dual-language children will make the typical errors found in monolingual comprehension of object RCs: Reversal Errors in Italian and Embedded Errors in Mandarin. Third, negative influence from Italian to Mandarin may be observed in the older group, where Reversal Errors may be evident in Mandarin. Becoming more familiar (and dominant) with Italian, our dual-language children will also be more familiar with the fact that the SVO order is typical of subject RCs in Italian and this will lead them to analyze a Mandarin object RC as a subject RC.

With respect to our third research aim, we expect to observe a delay in the comprehension of subject RCs in Mandarin as compared with that in Italian. As introduced earlier, it has been found that subject head-initial RCs are almost at ceiling in monolingual 3-year-old Italian-speaking children (and this holds for several other languages with head-initial RCs), while ceiling performance on subject head-final RCs is reached only at age 7 in monolingual Mandarin-speaking children. This different developmental pattern seems to indicate that head-final RCs may have an additional component of difficulty not present in head-initial RCs. Dual-language children are an ideal group of children to examine this conjecture.

## Materials and Methods

### Participants

Thirty-seven Mandarin–Italian dual-language children participated in the study in two groups: younger children (*N* = 19, *M* = 6;2, age range: 5;3–6;11) and older children (*N* = 18, *M* = 8;4, age range: 7;6–8;11). Data from three additional children were collected, but excluded because they did not finish all the tests reported below.

The children were recruited from the Chinese community in Milan, Italy. The criteria for the selection of the children were the following: no history of language impairment or hearing loss, regular exposure to Mandarin from birth, intensive and regular exposure to Italian starting in nursery or kindergarten, and use of both languages daily. After an initial screening done by the first author together with parents and teachers, all the parents of selected children completed a questionnaire that was an adaptation of the one used in Kidd et al. (2015) and the UBiLEC (Unsworth, 2013; Unsworth et al., 2014) to measure children’s language use and background. They were asked to indicate: (i) whether their child was born in Italy (if not, when they arrived in Italy); (ii) the amount of time per year they had visited China since birth; (iii) the first time they attended Italian schools (and how long for); (iv) the average amount of time the child spent in Mandarin- and Italian-speaking environments; (v) how often the child spoke each language at home on a 5-point scale (1 = never, 2 = rarely, 3 = sometimes, 4 = often, 5 = always); (vi) how well the child understood each language on a 7-point Likert scale (1 = poor, 7 = excellent); and (vii) whether they were able to read Chinese (if so, judge their literacy level compared with age-matched children in China). See Table 1 for participant characteristics.

**TABLE 1:** Mean age, summaries of language experience, and performance on PPVT.

	Younger Mean (*SD*)	Older Mean (*SD*)
Age^a^	74.42 (6.29)	100.33 (5.40)
Age of first exposure to Italian^a^	37.21 (3.28)	40.33 (8.17)
Length of exposure to Italian^a^	37.26 (5.74)	59.33 (9.03)
Time spent in Mandarin-speaking environment^b^	37.03 (15.92)	37.28 (15.37)
Time spent in Italian-speaking environment^b^	32.11 (10.99)	39.33 (9.16)
Frequency of speaking Mandarin^c^	3.79 (0.98)	3.89 (1.02)
Frequency of speaking Italian^c^	2.74 (0.93)	3.06 (1.11)
Rating of Mandarin comprehension^d^	5.05 (1.68)	5.00 (1.94)
Rating of Italian comprehension^d^	3.95 (1.68)	5.28 (1.49)
Mandarin PPVT^e^	40.68 (19.30)	83.89 (34.59)
Italian PPVT^e^	41.90 (18.84)	83.44 (31.14)

*^a^Age, age of first exposure to Italian, and length of exposure to Italian are expressed in months; ^b^time spent in Mandarin- or Italian-speaking environments are expressed in hours per week; ^c^frequency of speaking Mandarin and Italian at home is expressed on a 5-point scale; ^d^rating of how well children understood Mandarin and Italian is expressed on a 7-point Likert scale; ^e^PPVT are expressed in raw scores (and standard deviation).*

All the children reported in the present study were born in Italy and grew up in households where Mandarin was spoken. All the parents were Mandarin native speakers and predominately used their native language with their children; the children also predominately spoke Mandarin with their parents. In addition, the children had access to Mandarin through other native Mandarin speakers in their extended social networks in Italy and from short visits to China. They attended weekend classes and summer camps in a Chinese cultural center in Milan where Mandarin was the medium of instruction. They were all able to read Chinese as they learned it in Mandarin classes, and according to their parents’ judgment, their literacy was much lower than that of age-matched children in China. All the children went to Italian nursery or kindergarten between age 2 and age 4, and all were educated in Italian public schools. The younger group were regularly exposed to Italian at the mean age of 37.21 months and, at the time of testing, had been exposed to Italian for 37.26 months. The older group was regularly exposed to Italian at the mean age of 40.33 months and had been exposed to Italian for 59.33 months. There was no significant difference between groups in terms of age of first exposure to Italian, while there was a significant difference in terms of length of exposure [*t*(35) = −8.92, *p* < 0.001]. The older group had been exposed to Italian 2 years longer than the younger group.

In addition, there were significant differences between groups on the average amount of time the children spent in the Italian-speaking environment at the time of the study [*t*(35) = −2.17, *p* < 0.05] and on parents’ rating of how well their children understood Italian [*t*(35) = −2.54, *p* < 0.05]. No other difference was found. Overall, the children in the older group spent more time in an Italian-speaking environment than the children in the younger group, and the older children’s comprehension of Italian was rated higher than that of the younger children.

Then, we compared the language experience of the two groups. First, there was no significant difference within each group regarding the average amount of time the children spent in a Mandarin- or Italian-speaking environment. Second, children spoke Mandarin more frequently than Italian at home: the younger group [*t*(18) = 3.40, *p* < 0.01] and the older group [*t*(17) = 3.40, *p* < 0.05]. Third, parents of the younger group rated their children’s comprehension of Mandarin as higher than their comprehension of Italian [*t*(18) = 2.02, *p* = 0.0503]; no difference was observed in the older group.

Children’s linguistic competence was also measured using the Peabody Picture Vocabulary Test (PPVT; Dunn and Dunn, 1981; Stella et al., 2000). The difference between children’s Mandarin PPVT and Italian PPVT scores was not significant in the younger group or the older group. This is a hint that the children’s vocabulary knowledge was balanced.

### Materials

We examined the comprehension of subject and object RCs in Mandarin and Italian using a character-sentence matching task. Two sets of the materials were constructed, each with a Mandarin and an Italian version. Each set of the materials consisted of 8 subject RCs and 8 object RCs, as exemplified in (5a-b) for Mandarin RCs, and in (6a-b) for Italian RCs.


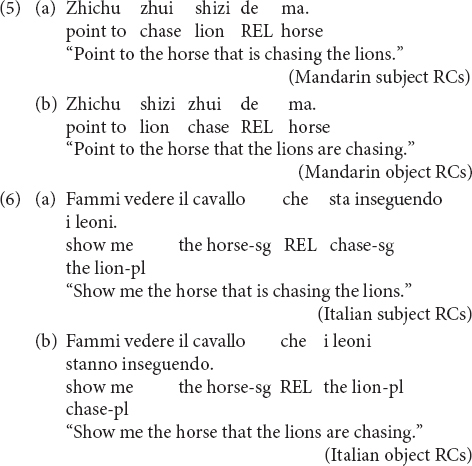


**FIGURE 1:**
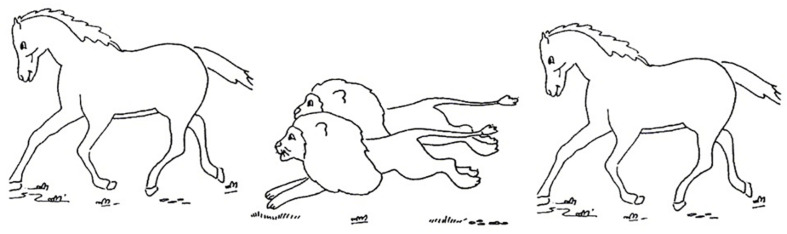
An experimental picture used in the Mandarin and Italian experiments on RC comprehension. This picture can be used to test comprehension of both subject RCs and object RCs such as (5) and (6).

The task consisted of 16 black and white pictures with the same structure (i.e., one animal X on the left, a pair of animals Y in the middle, and another X on the right). The pictures depicted eight actions including bite, chase, follow, hit, push, smell, splash, and wipe. They were presented an equal number of times, i.e., each action appears in the four RCs exemplified in (5) and (6). To avoid priming effects, each picture was used only once in each version of the test. Figure 1 is a sample of the experimental pictures. For sentences (5a) and (6a) the correct answer is the horse on the right, and for sentences (5b) and (6b) the correct answer is the horse on the left.

In addition, there were 8 filler sentences involving intransitive verbs (e.g., *sleep*) or actional irreversible verbs (e.g., *drink*), and 3 practice items. In total, there were 27 items in each version of the RC test (see Supplementary Material for the materials). All the sentences were recorded by female native speakers of Mandarin or Italian.

### Procedure

Before testing, written consent and the questionnaires were collected from parents. The study was approved by the Ethics Committee of the University of Milano-Bicocca according to the standards of the Helsinki Declaration (1964).

Participants were tested individually in Chinese schools or university. They were given the Mandarin tests and the Italian tests in two sessions, with a 1-or-2-week interval between the sessions. The order of testing language was counterbalanced: half of the children first completed the tests in Mandarin and then in Italian, and half vice versa. The experimental materials of the tests were presented on a laptop using Microsoft PowerPoint. Each task was explained to the children. For the RC tests, each child was instructed to point to the character referred to in the sentence, and was given practice items to familiarize themselves with the task.

### Scoring and Error Coding

In the RC comprehension tests, the dependent variable was the proportion of accurate responses, namely, the accuracy in identifying the correct character. When participants did not choose the correct character(s), we coded the response as an Error. Errors were labeled as Reversal Errors and Embedded Errors.^3^

Consider (5b), i.e., *the horse that the lions are chasing*, and Figure 1. A Reversal Error consisted of choosing the horse on the right, i.e., *the horse that is chasing the lions*, rather than the horse on the left. Here, the theta-roles are reversed, i.e., in (5b) the relative head *the horse* is a Patient, but the child interpreted it as an Agent. An Embedded Error was coded when children pointed to the middle characters corresponding to the embedded NP in the RC. For example, for the sentence *the horse that the lions are chasing*, children pointed to the characters in the middle, i.e., *the lions*, which is the subject of the RC.

### Statistical Analysis

In this study, we used R (R Core Team, 2018) and *lme4* (Bates et al., 2013) to perform linear mixed-effects analyses of the relationship between different fixed factors. Models were constructed with a maximal random effects structure and were successively simplified when they failed to converge (Barr et al., 2013). Language (Mandarin vs. Italian), Sentence Type (subject RCs vs. object RCs), and Group (younger vs. older) were categorical variables, while Age was a continuous variable. Reference levels were Italian for Language, object RCs for Sentence Type, and older for Group. For simplicity, we mainly report the details in each analysis for significant effects. In addition, we explored individual differences and error types, using Pearson’s Chi-squared tests with Yates’ continuity correction and Poisson regression models, respectively. In the end, correlations among measures of linguistic background and RC comprehension were computed to assess the relation between language experience and RC comprehension.

## Results

We report the results of RC comprehension, with the order of the analyses of correct responses, individual performance, the error analyses, and the correlation analyses.

### Correct Responses

**FIGURE 2:**
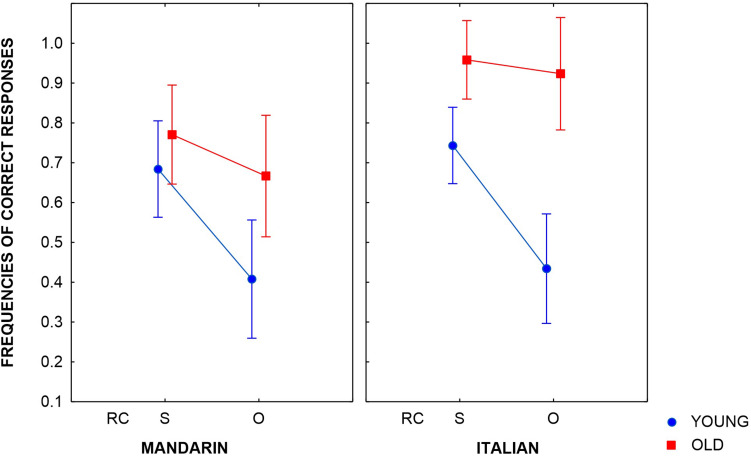
Correct responses of Mandarin–Italian dual-language children in the comprehension of subject and object RCs (bars indicate a standard error). Two groups of children participated in the study: younger (*M* = 6;2) and older (*M* = 8;4). On the *X*-axis, we have the type of relative clause (RC): subject (S) and object (O) RCs. On the *Y*-axis, we have frequencies of correct responses.

Figure 2 reports the frequencies of correct responses in the younger and older groups from dual-language children. The younger children comprehend RCs less well than the older children did and they displayed a clear advantage in the comprehension of subject RCs with respect to object RCs. In addition, comprehension of Italian RCs was higher than that of Mandarin RCs in the older group.

**FIGURE 3:**
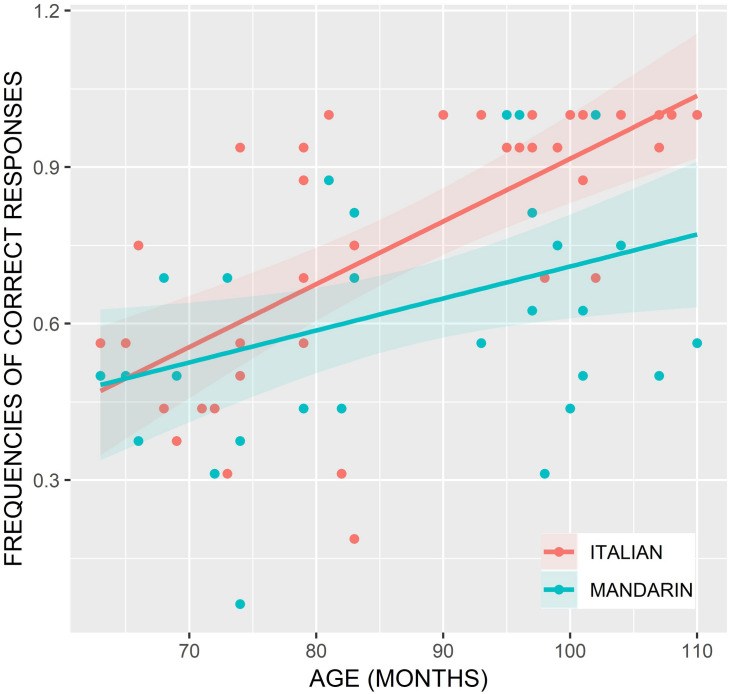
Graph of the relationship between age (in months) and percentage of correct responses in the comprehension of Mandarin and Italian RCs.

We first analyzed the dual-language children’s correct responses, considering Age a continuous variable, and Language (Mandarin vs. Italian) and Sentence Type (subject RCs vs. object RCs) as categorical variables. As random effects, the models presented by-subject and by-item intercepts. There were significant effects of Age, Language, and Sentence Type (see Table 2), and no interaction between Language and Sentence Type. We visualized the main findings in Figure 3. Both lines have a positive slope, indicating that accuracy increased with age, but the slope of the Mandarin RC line is less steep than that of the Italian RC line, indicating that accuracy of Italian RC comprehension increased faster over age than that of Mandarin RC comprehension.

**TABLE 2:** Summary of the significant fixed effects in the mixed-effects model (*N* = 1184, log likelihood = −606.5) in the RC comprehension.

	Estimate	SE	Wald *Z*	*p*
(Intercept)	−3.81	1.06	−3.60	−0.000 ***
Age	0.05	0.01	4.54	−0.000***
Language	−0.75	0.19	−3.89	−0.000 ***
Sentence type	1.19	0.22	5.38	−0.000 ***

Age is expressed in months; reference level for Language = Italian; reference level for Sentence Type = object RCs; ***p < 0.001.

To better understand the dual-language children’s performance, we further analyzed their correct responses, adopting a factorial approach to their age (i.e., the younger and the older groups). Group (younger vs. older), Language (Mandarin vs. Italian), and Sentence Type (subject RCs vs. object RCs) were initially entered into a factorial model, and all significantly contributed to the model fit [χ^2^(3) = 107.25, *p* < 0.001]. Notably, there were significant Group and Language effects qualified by a Group × Language interaction. See Table 3 for a summary of the significant effects of the full analysis.

**TABLE 3:** Summary of the significant fixed effects in the mixed-effects model (*N* = 1184, log likelihood = −587.2) in the RC comprehension.

	Estimate	*SE*	Wald *Z*	*p*
(Intercept)	2.90	0.41	7.14	−0.000***
Group	−3.18	0.49	−6.48	−0.000***
Language	−2.03	0.38	−5.38	−0.000***
Group × Language	1.91	0.45	4.22	−0.000***

Reference level for Group = older; reference level for Language = Italian; ***p < 0.001.

To explore the interaction, we analyzed each group separately and each language separately. For each group, we analyzed the data with a mixed-effects model including Language (Mandarin vs. Italian) and Sentence Type (subject RCs vs. object RCs) as fixed factors, and by-subject and by-item random intercepts. We analyzed each language separately and used a mixed-effects model including Sentence Type (subject RCs vs. object RCs) and Group (younger vs. older) as fixed factors, and by-subject and by-item random intercepts.

#### The Younger Group

Only the main effect of Sentence Type was significant (β = 1.48, Wald *Z* = 5.63, *p* < 0.001). The results suggest that the younger dual-language children comprehended subject RCs significantly better than object RCs, in line with previous findings on monolingual children that there is a subject/object asymmetry in the comprehension of RCs (at least up to a certain age).

#### The Older Group

Only the main effect of Language was significant (β = −2.10, Wald *Z* = −5.43, *p* < 0.001). These results prove that after about 5 years of exposure to Italian, the dual-language children comprehended Italian RCs significantly better than Mandarin RCs. Comprehension of Italian RCs is almost at ceiling. As it happens, in older monolingual children no subject/object asymmetry is evident (at least with this type of task).^4^

#### Mandarin

We found main effects of Sentence Type (β = 0.64, Wald *Z* = 2.19, *p* = 0.03) and Group (β = −1.43, Wald *Z* = −3.01, *p* = 0.003), and a marginally significant interaction between them (β = 0.73, Wald *Z* = 1.84, *p* = 0.07). The results suggest that the older group performed better than the younger group, as we had already established in the general analysis, and this was especially true for object RCs. As the inspection of Figure 2 reveals, the increase in subject RCs is numerically much smaller than in object RCs, which, added to the marginally significant interaction, suggests that the older group’s improvement was mainly observed in object RCs.

#### Italian

Only the main effect of Group was significant (β = −3.52, Wald *Z* = −5.58, *p* < 0.001). These results suggest that the older group performed better than the younger group, as we also established in the general analysis.

To wrap up, the younger group displayed a clear subject/object asymmetry in both languages. The performance of younger Mandarin–Italian dual-language children is similar in the two languages, as far as RC comprehension is concerned. This similarity is remarkable. Although these children had been exposed to Italian for about 3 years, their level was comparable to that of their Mandarin. Recall that this similarity was also evident in their PPVT scores in the two languages. The younger group understood RCs less well than the older group, both in Mandarin and in Italian, that is, with more exposure, comprehension of RCs improved.

Interestingly, the older group displayed a higher comprehension of RCs in Italian than in Mandarin; that is, their improvement was higher for the majority language. In fact, in Italian, their performance was at ceiling. Notice that the older group did not display the subject/object asymmetry in either of their two languages. Recall that this asymmetry typically disappears when children grow older and is not evident in adults, at least if comprehension is measured with the character-sentence matching task (see footnote 4). This lack of asymmetry in Italian is understandable, as children are at ceiling. It is more surprising in Mandarin, as children are not at ceiling. In general, during development we observe an asymmetry until children reach ceiling performance and often this ceiling performance is reached first for subject RCs. This was not the situation for our children. Together with the observation that in Mandarin, object RCs improved more than subject RCs from the younger to the older group (the marginal significant interaction), this lack of asymmetry suggests that Mandarin subject RCs display some aspects of complexity, as do Mandarin object RCs.

### Individual Performance

We further ran an individual analysis by examining the number of participants performing above chance in each condition. In the task, participants have to choose one character out of three and answer 8 items in each condition. According to the binomial distribution, the performance was considered as above chance when six responses (out of eight) in each condition were correct. Table 4 reports the percentages of participants performing above chance level in comprehending Mandarin and Italian RCs.

**TABLE 4:** Percentages of participants who performed above chance (i.e., six correct responses out of eight) in the comprehension of subject and object RCs.

Group	Mandarin		Italian	
	Subject RCs	Object RCs	Subject RCs	Object RCs
Younger	68% (13/19)	32% (6/19)	63% (12/19)	26% (5/19)
Older	61% (11/18)	50% (9/18)	94% (17/18)	94% (17/18)

We explored these results by running Pearson’s Chi-squared tests with Yates’ continuity correction, comparing the number of children who performed above chance level with the number of children that did not.

We first compared the number of children who performed above chance in the two languages, according to their age. In the younger group, there was no significant difference between the two languages in subject RCs or in object RCs. This result is in line with the previous analysis showing no language difference in the younger group. In the older group, a significant difference between the two languages was evident, in both subject [χ^2^(1) = 4.02, *p* = 0.045] and object RCs [χ^2^(1) = 6.78, *p* = 0.009].

In addition, we counted the number of participants who performed above chance in both subject and object RCs. In the case of Mandarin, 16% (3 out of 19) of the children in the younger group and 44% (8 out of 18) of those in the older group did so, but the difference between the two groups did not reach significance [χ^2^(1) = 2.39, *p* = 0.12]. In the case of Italian, 21% (4 out of 19) of the children in the younger group and 89% (16 out of 18) of those in the older group did so. This difference was significant [χ^2^(1) = 14.50, *p* < 0.001].

To sum up, the results of individual analyses confirm that the older dual-language children comprehended subject and object RCs in Italian better than those in Mandarin, although they had been exposed to Mandarin from birth and continued to use it. In addition, more children in the older group performed above chance on both RC types in Italian than the younger group. This was not the case in Mandarin; the number of children performing above chance across the two groups did not significantly differ. It appears clear that both at the group and individual levels, improvement is more evident in Italian than in Mandarin. In addition, the improvement in Mandarin is more evident in object RCs.

### Error Analyses

We further investigated what children did when they failed to understand RCs, by examining the distribution of errors. Table 5 summarizes means and standard deviation of error types (i.e., Reversal Errors and Embedded Errors) for each group in the comprehension of RCs in the two languages.

**TABLE 5:** Means (and standard deviation) of incorrect responses in the comprehension of subject and object RCs (means are calculated over the total number of responses).

Group	Mandarin		Italian	
	Subject RCs	Object RCs	Subject RCs	Object RCs
**Younger**				
Correct response	0.68 (0.30)	0.41 (0.32)	0.74 (0.27)	0.43 (0.38)
Reversal error	0.13 (0.23)	0.18 (0.21)	0.12 (0.18)	0.36 (0.33)
Embedded error	0.19 (0.18)	0.41 (0.36)	0.14 (0.16)	0.21 (0.24)
**Older**				
Correct response	0.77 (0.21)	0.67 (0.32)	0.96 (0.10)	0.92 (0.16)
Reversal error	0.17 (0.21)	0.21 (0.24)	0.03 (0.09)	0.06 (0.11)
Embedded error	0.06 (0.12)	0.12 (0.24)	0.01 (0.04)	0.02 (0.06)

We counted numbers of each type of error that each child made, and treated it as a count variable to run a Poisson regression model.

Let us first consider the errors that the younger group made. In subject RC comprehension, no difference between Reversal and Embedded Errors was found in Mandarin or in Italian (β = −0.34, Wald *Z* = −1.15, *p* = 0.25, and β = −0.15, Wald *Z* = −0.48, *p* = 0.63, respectively). In the comprehension of Mandarin object RCs, Embedded Errors were significantly more frequent than Reversal Errors (β = −0.85, Wald *Z* = −3.68, *p* < 0.001). By contrast, in the comprehension of Italian object RCs, the opposite pattern was found, namely, Reversal Errors were significantly more frequent than Embedded Errors (β = 0.52, Wald *Z* = 2.35, *p* = 0.019).

Second, consider the errors made by the older group. In subject RC comprehension, Reversal Errors were significantly more frequent than Embedded Errors in Mandarin (β = 0.98, Wald *Z* = 2.51, *p* = 0.012), but no significant difference between the two error types was observed in Italian (β = 0.69, Wald *Z* = 0.80, *p* = 0.42). In the comprehension of Mandarin object RCs, Reversal Errors were more frequent than Embedded Errors in Mandarin (β = 0.60, Wald *Z* = 2.00, *p* = 0.047), while in the comprehension of Italian object RCs, no significant difference between the two types of errors was evident (β = 0.98, Wald *Z* = 1.45, *p* = 0.15).

To sum up, when the younger children were not able to comprehend subject RCs, they made Embedded or Reversal Errors both in Mandarin and in Italian. In other words, they chose another character randomly. When they did not comprehend object RCs, they were more likely to make Embedded Errors in Mandarin, but Reversal Errors in Italian. Notice that, as stated earlier, given the different structures of Mandarin and Italian object RCs, the two different types of errors led to the choice of the first NP heard in both languages. As for the older children, they still had difficulties in the comprehension of Mandarin RCs. Unlike the younger children, they were more likely to make Reversal Errors than Embedded Errors in Mandarin subject RCs and object RCs.

### Correlation Analyses

Several correlations were found between the comprehension of Mandarin RCs and Italian RCs. The children who were better at understanding object RCs in Italian were better at doing the same thing in Mandarin. This holds true both for the younger (Pearson correlation coefficient = 0.60, *p* < 0.01) and the older groups (Pearson correlation coefficient = 0.48, *p* < 0.05).

In addition, we found a correlation between the comprehension of Italian RCs and the length of exposure to Italian (Pearson correlation coefficient = 0.63, *p* < 0.001), which holds true for both subject RCs (Pearson correlation coefficient = 0.43, *p* < 0.01) and object RCs (Pearson correlation coefficient = 0.57, *p* < 0.001).

Interestingly, we found a correlation between the comprehension of Mandarin RCs and the frequency with which children spoke Mandarin in the home (Pearson correlation coefficient = 0.44, *p* < 0.01). In particular, this holds true for subject RCs in the younger group (Pearson correlation coefficient = 0.66, *p* < 0.01) and there is a marginal significant difference for object RCs in the older group (Pearson correlation coefficient = 0.45, *p* = 0.06). By contrast, we found a correlation between the comprehension of Italian RCs and the average amount of time the child spent in an Italian-speaking environment (Pearson correlation coefficient = 0.37, *p* < 0.05). This holds particularly true for object RCs in the younger group (Pearson correlation coefficient = 0.46, *p* < 0.05). The results indicate that children’s comprehension of Mandarin RCs is more related to how often they spoke the language, while their comprehension of Italian RCs is more related to the average amount of time they spent in an Italian-speaking environment.

Moreover, in the older group, we found a correlation between comprehension of Mandarin object RCs and Mandarin vocabulary score (Pearson correlation coefficient = 0.51, *p* < 0.01). By contrast, we did not observe any significant correlations between children’s comprehension of Italian subject and object RCs and their vocabulary knowledge in Italian, suggesting that comprehension of Italian RC does not depend on vocabulary knowledge. Recall that the level of Italian and Mandarin vocabulary were similar in the older group, but their comprehension of Italian RCs was more advanced than that of Mandarin RCs.

## Discussion

Our findings revealed a complex picture of RC comprehension in dual-language children speaking Mandarin and Italian. For convenience, we discuss the findings in distinct subsections.

### The Role of Length of Exposure to the Majority Language

Regarding our first aim, we found that the length of exposure to the majority language contributed to shaping comprehension of RCs and this was evident in both groups, although in different ways.

First, the younger group comprehended subject RCs better than object RCs in both of their languages. This was the only significant effect found in the younger group, suggesting that 3 years of exposure to Italian were enough to put these children at the same level as they were in their Mandarin, as far as RC comprehension is concerned. We attribute this subject RC advantage to subject RCs being structurally simpler than object RCs in both languages. The dependency between the relative head and its copy is shorter in subject RCs than in object RCs. In other words, nothing intervenes between the two in the former case, while it does in the latter case; specifically, the subject intervenes in the dependency. Based on a corpus analysis of Mandarin child-directed speech from CHILDES, Tsoi et al. (2019) attributes the Mandarin subject RC advantage to the frequency of RC-like structures (e.g., possessive structures). In Mandarin, *de* has different functions beyond being a relativizer. Tsoi et al. (2019) found that the subject RC-like structures [VN de (N)] are more frequent than the object RC-like structures [NV de (N)]. However, as they notice, if RCs are extrapolated from these RC-like structures, the opposite holds: object RCs are more frequent than subject RCs. Accordingly, they claim that frequency of information matters, but various levels of frequency may be differently relevant. We are skeptical about this conclusion, as there are several studies on RC comprehension, which found that Italian passive object RCs (i.e., an object RC turned into a subject one through passivization), like in (7a), are easier to acquire than active object RCs, as in (7b) (see Contemori and Belletti, 2014; Belletti and Guasti, 2015; Arosio et al., 2017).


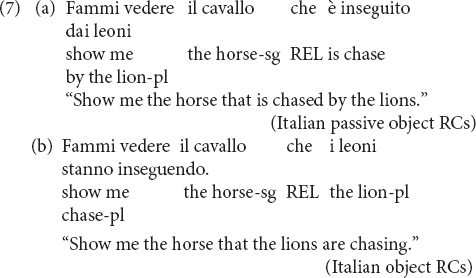


Interestingly, in corpora, passive object RCs are extremely rare and less frequent than object RCs (Belletti and Chesi, 2011). The reason why passive object RCs are easier than active ones is that they are structurally simpler, either in terms of distance or in terms of lack of intervention effects.

It is worth pointing out that our results converge with those reported by Tsoi et al. (2019) in showing that the subject RC advantage in Mandarin dual-language children as found in our younger group is not due to influence from a language with head-initial RCs, be it English or Italian. However, this influence seems active in multilingual children speaking Cantonese, based on Kidd et al. (2015) and Chan et al. (2017).

Second, the older group had a better comprehension of RCs in Italian than in Mandarin. We conclude that after about 5 years of intensive exposure to Italian (and 2 years of formal instruction), better performance is found in the majority language than in Mandarin. In the older group, the subject/object asymmetry in the comprehension of RCs disappeared in Italian, because children were at ceiling. It also disappeared in Mandarin, but in this language, we observed that the older group of children improved in the comprehension of object RCs slightly more than in the comprehension of subject RCs, although this interaction was only marginally significant. The comprehension of Mandarin RCs was poorer than that of Italian ones. Thus, concerning our first aim, we can say that after 3 years of immersion in the majority language children catch up with their minority language (the younger group) and with 5 years of exposure and formal education they attain native-like competence and are similar to the monolingual 7-year-olds studied in Adani (2011), as far as comprehension of RCs is concerned. Concerning the younger group, it is quite remarkable that children reach the same level of comprehension in Italian as in Mandarin, in spite of just 3 years of exposure to Italian, in contrast with 6 years to Mandarin. No correlation between vocabulary comprehension and RC comprehension emerges in Italian, suggesting that the two competencies develop separately in Italian. In fact, while in the older group the comprehension of Italian RCs was at ceiling, their vocabulary comprehension corresponded to a standard score of 79 (raw scores = 83.44) based on a monolingual Italian norm. This is not surprising, as learning the vocabulary depends on the contexts of use of a language (if Italian is rarely spoken at home, words used in the family context may be absent). In contrast, once the RC structure is acquired, there is nothing more to do.^5^

With respect to monolingual Mandarin-speaking age-matched children (who achieve adult levels at age 8), based on Hu et al. (2016), our older group performed less well numerically. At the same time, we found a correlation between the frequency with which children spoke Mandarin in the home and the comprehension of Mandarin RCs, suggesting that active use of a language is important to letting the language grow. We also uncovered a correlation between the comprehension of RCs in the two languages: children who were better in one language were also better in the other language, suggesting reciprocal support. Thus, using two languages, even with structures that are quite different, as RCs, promotes comprehension of these structures in both languages. This is a hint that at some levels Mandarin and Italian RCs share the same representational structure.

### Bidirectional Influence

We turn now to our second aim. Beyond the positive and general reciprocal support, the bidirectional influence was especially evident in the error analysis. From the quantitative point of view, the younger group made a similar number of errors in the comprehension of subject RCs in their two languages: 32% in Mandarin and 26% in Italian (Embedded and Reversal Errors together). This result is in line with what the monolingual Mandarin-speaking children tested by Hu et al. (2016) did at the age of 6 years: they produced 24% errors. However, there is a sharp contrast with what the monolingual Italian-speaking children studied by Adani (2011) did: they only made 9% of errors at age 3 (and 4% at age 6). This contrast may be due to the fact that although dual-language children were regularly exposed to Italian for about 3 years (i.e., the same time as the Italian monolinguals in Adani, 2011), they received less Italian input than monolinguals. Alternatively, the contrast may be a hint that acquiring RCs with opposite directionality is challenging. It is possible that children, at least in the initial stage of exposure to Italian, have trouble dealing with two structures displaying dependencies that go in opposite directions (the relative head precedes the gap in Italian and follows it in Mandarin). If it is the different directionality that matters, we expect children acquiring an L2, in which RCs have the same word order, not to experience difficulties in the comprehension of subject RCs. On the contrary, if it is insufficient input that matters, then we expect these children to experience the same difficulties observed in our Mandarin–Italian speaking children. Another alternative is that the “SV *de* O” structure of Mandarin object RCs misleads children as it is superficially similar to that of Italian subject RCs with the structure “S that VO.” Under this view, the high number of errors with Italian subject RCs could be a manifestation of negative influence from Mandarin. This conjecture is supported by a qualitative analysis of the errors in subject RCs. Our Mandarin–Italian dual-language children, like monolingual Mandarin-speaking children (Hu et al., 2016), Mandarin–English dual-language children (Tsoi et al., 2019), and Cantonese–English–Mandarin trilingual children (Chan et al., 2017), made Embedded and Reversal Errors in Mandarin when they had to comprehend a subject RC. Interestingly, they did the same in Italian, unlike monolingual Italian-speaking children, who, according to Adani (2011), made very few Reversal Errors. We interpret the presence of both Reversal and Embedded Errors in Italian as an indication that the Mandarin–Italian dual-language children were confused and randomly chose either the first NP or the last NP heard, as the monolingual Mandarin-speaking children did in Hu et al. (2016).

The errors displayed in the comprehension of object RCs do not seem to result from any bidirectional influence between the two languages in the younger group. The types of errors are similar to those made by the monolingual Mandarin-speaking children (Hu et al., 2016) and the monolingual Italian-speaking children (Adani, 2011): Embedded Errors in Mandarin and Reversal Errors in Italian. Embedded Errors were also found in Mandarin–English dual-language children (Tsoi et al., 2019) and Cantonese–English–Mandarin trilingual children (Chan et al., 2017) in the comprehension of Mandarin object RCs. As we said, given the structure of RCs in the two languages, an Embedded Error in Mandarin amounts to choosing the first NP heard as the Agent of the action and similarly for Reversal Errors in Italian. Thus, this error may be the result of a common strategy in the two languages: the first NP is the chosen referent and Agent of the action.

The influence between the two languages re-emerges in the older group. First, the older group was almost perfect in Italian, behaving as monolingual Italian-speaking children at the age of 7. They still made many errors in the comprehension of Mandarin subject and object RCs. However, Embedded Errors were significantly less frequent than Reversal Errors both in subject and object RCs, that is, the older children preferred reversing the thematic roles. This shift in the type of error was not observed in the monolinguals by Hu et al. (2016), where Embedded Errors were the most common type in object RC comprehension for all age groups investigated (from 3 to 7 years). This finding can be attributed to negative influence from Italian to Mandarin. As we said earlier, Italian subject RCs display the order “S that VO,” which superficially corresponds to the surface order of Mandarin object RCs. This similarity may mislead dual-language children toward a subject RC analysis of Mandarin object RCs.

Thus, the influence of one language on the other is evident in three ways. There is negative influence from Mandarin to Italian in the younger group (evident in the comprehension of Italian subject RCs), negative influence in the older group from Italian to Mandarin (evident in the error analysis concerning Mandarin object RCs), and reciprocal support in that better comprehension of RCs in one language correlated with better comprehension in the other one.

### Is There a Delay in the Comprehension of Head-Final Subject Relative Clauses?

Our third aim was to gain insight into the delay in the comprehension of subject RCs in Mandarin with respect to languages with head-initial RCs. Although subject RCs are easier to understand than object RCs, they were challenging for the younger children and elicited 32% errors; so were subject RCs in Italian (26% errors), which we attributed to the influence from Mandarin and/or lack of sufficient input in Italian. This evidence notwithstanding, we may remark that the younger children had been exposed to Mandarin, from birth, for 6 years, and to Italian for half of that time, though they had less exposure to each language compared with monolinguals. Hence, the fact that the percentages of errors are almost the same in the two languages suggests that Mandarin subject RCs are more challenging than Italian ones. This observation is further corroborated by the 5–6-year-old trilingual children, whose rate of correct responses for Cantonese (L1) and Mandarin (L3) subject RCs was 29% and 34%, respectively (Chan et al., 2017). For their L2 English, this rate was 90%. For the Mandarin–English bilinguals (*M* = 6;1), the rate of correct responses was 48% in Mandarin and 80% in English (Tsoi et al., 2019). Taken together, these findings support the conclusion that subject head-final RCs contain some elements of complexity, which are not present in subject head-initial RCs.

Earlier, we attributed the subject advantage to their structural simplicity. Under the explanation that we adopted, this simplicity consists in the fact that in subject RCs nothing intervenes hierarchically between the relative head and its copy (Friedmann et al., 2009). Along these lines, what may be challenging in head-final subject RCs is the fact that the object intervenes linearly between the relative head and its backward copy (Hu et al., 2016). Linear intervention is less disruptive than structural intervention, as shown in Franck et al. (2006), hence the subject advantage also evident in Mandarin. However, it contributes some penalty, which causes trouble for children acquiring head-final RCs. This delay notwithstanding, we have to acknowledge that the older group is weak in its comprehension of Mandarin RCs and this is likely due to the lower input to which they are exposed. We mentioned that RCs are explicitly taught at school in Italy. This may suggest that similar teaching would be helpful in Mandarin classes, as it would exploit the kind of competence that is already developed in Italian classes.

## Conclusion

We replicated the subject advantage in Mandarin (younger group), as found in several other studies. We showed that the length of exposure to the majority language affects comprehension of RCs, even in a situation in which the minority language is supported both at the oral and the written level. In particular, the time spent in the majority language environment correlated with RC comprehension. Nevertheless, we found reciprocal support between the two languages in the comprehension of RCs, suggesting that double language use must be sustained. The use of two languages leads to bidirectional influence, and this is evident in the errors that are made as a function of length of exposure: the younger and the older group behaved differently, likely due to which language was more frequently used. Finally, the penalty that subject head-final RCs seems to have with respect to subject head-initial RCs is likely due to linear intervention, a process less disruptive than structural intervention, but that still affects comprehension.

## Data Availability Statement

The datasets generated for this study are available on request to the corresponding author.

## Ethics Statement

The studies involving human participants were reviewed and approved by University of Milano-Bicocca. Written informed consent to participate in this study was provided by the participants’ legal guardian/next of kin.

## Author Contributions

SH and MG conceived the research question, developed the experimental task, and drafted the manuscript. SH recruited and tested the participants and performed the statistical analyses. FC developed the experimental task and tested the participants. All authors contributed to the article and approved the submitted version.

## Conflict of Interest

The authors declare that the research was conducted in the absence of any commercial or financial relationships that could be construed as a potential conflict of interest.

## Footnotes

1 Our children started to learn the L2 between 2 and 4 years of age. Some authors refer to these children as bilingual, while for others they are early L2 learners. To avoid entering into this debate, we used the term “dual-language children” in line with some articles in the literature to refer to children who use two languages, as this is a more neutral term.

2 In Italian, object RCs can occur in another order, i.e., the object-verb-subject (OVS) order. As in (i), the embedded subject *il cane* “the dog” follows the verb *colpisce* “hits,” with which it agrees in number. For the relevant discussion, see Adani (2011).

3 What we call an Embedded Error is called a Middle Error in Adani (2011). It should be noticed that the task of our study has three choices as in Adani (2011), but differs from the task which has four choices (e.g., Hu et al., 2016; Tsoi et al., 2019).

4 A subject RC advantage is still observed in adults and older children if reading times are measured (see the comparison of the comprehension of RCs by children and adults in Guasti et al., 2018).

5 The correlation between vocabulary and object RC comprehension was found in Mandarin, though. This suggests that Mandarin and Italian do not develop in parallel, although reciprocal support is found between the comprehension of RCs in the two languages.
