# Supplementary material for: Mandarin–Italian Dual-Language Children’s Comprehension of Head-Final and Head-Initial Relative Clauses
Source: Front Psychol. 2020 Aug 11;11:1379. doi: 10.3389/fpsyg.2020.01379 (PMC7431764; doi:10.3389/fpsyg.2020.01379)
Supplement: Supplementary file 1 [file Data_Sheet_1.docx]

**Supplementary Material**

# Experimental Materials

1. bite
2. the lion/the camel

咬骆驼的狮子/il leone che sta mordendo i cammelli

“the lion that is biting the camels”

骆驼咬的狮子/il leone che i cammelli stanno mordendo

“the lion that the camels are biting”

1. the monkey/the pig

咬猪的猴子/la scimmia che sta mordendo i maiali

“the monkey that is biting the pigs”

猪咬的猴子/la scimmia che i maiali stanno mordendo

“the monkey that the pigs are biting”

1. chase
2. the horse/the lions

追狮子的马/il cavallo che sta inseguendo i leoni

“the horse that is chasing the lions”

狮子追的马/il cavallo che i leoni stanno inseguendo

“the horse that the lions are chasing”

1. the goose/the rabbit

追兔子的鹅/l’oca che sta inseguendo i conigli

“the goose that is chasing the rabbits”

兔子追的鹅/l’oca che i conigli stanno inseguendo

“the goose that the rabbits are chasing”

1. follow
2. the bear/the rabbit

跟着兔子的熊/l’orso che sta seguendo i conigli

“the bear that is following the rabbits”

兔子跟着的熊/l’orso che i conigli stanno seguendo

“the bear that the rabbits are following”

1. the bear/the pig

跟着猪的熊/l’orso che sta seguendo i maiali

“the bear that is following the pigs”

猪跟着的熊/l’orso che i maiali stanno seguendo

“the bear that the pigs are following”

1. hit
2. the bear/the elephant

打大象的熊/l’orso che sta colpendo gli elefanti

“the bear that is hitting the elephants”

大象打的熊/l’orso che gli elefanti stanno colpendo

“the bear that the elephants are hitting”

1. the horse/the lion

打狮子的马/il cavallo che sta colpendo i leoni

“the horse that is hitting the lions”

狮子打的马/il cavallo che i leoni stanno colpendo

“the horse that the lions are hitting”

1. push
2. the bear/the elephant

推大象的熊/l’orso che sta spingendo gli elefanti

“the bear that is pushing the elephants”

大象推的熊/l’orso che gli elefanti stanno spingendo

“the bear that the elephants are pushing”

1. the monkey/the camel

推骆驼的猴子/la scimmia che sta spingendo i cammelli

“the monkey that is pushing the camels”

骆驼推的猴子/la scimmia che i cammelli stanno spingendo

“the monkey that the camels are pushing”

1. smell
2. the horse/the cow

闻牛的马/il cavallo che sta annusando le mucche

“the horse that is smelling the cows”

牛闻的马/il cavallo che le mucche stanno annusando

“the horse that the cows are smelling”

1. the cow/the sheep

羊闻的牛/la mucca che sta annusando le pecore

“the cow that is smelling the sheep”

闻羊的牛/la mucca che le pecore stanno annusando

“the cow that the sheep are smelling”

1. splash
2. the rabbit/the cock

喷公鸡的兔子/il coniglio che sta bagnando i galli

“the rabbit that is splashing the cocks”

公鸡喷的兔子/il coniglio che i galli stanno bagnando

“the rabbit that the cocks are splashing”

1. the dog/the turtle

喷乌龟的狗/il cane che sta bagnando le tartarughe

“the dog that is splashing the turtles”

乌龟喷的狗/il cane che le tartarughe stanno bagnando

“the dog that the turtles are splashing”

1. wipe
2. the cat/the rabbit

擦兔子的猫/il gatto che sta pulendo i conigli

“the cat that is wiping the rabbits”

兔子擦的猫/il gatto che i conigli stanno pulendo

“the cat that the rabbits are wiping”

1. the dog/the sheep

擦羊的狗/il cane che sta pulendo le pecore

“the dog that is wiping the sheep”

羊擦的狗/il cane che le pecore stanno pulendo

“the dog that the sheep are wiping”

# Filler Materials

1. 在花上的大蜜蜂/la grande ape sul bel fiore

“the large bee on the beautiful flower”

1. 在大树上的松鼠/lo scoiattolo sul grande albero

“the squirrel on the big tree”

1. 睡觉的猫/il gatto che sta dormendo

“the cat that is sleeping”

1. 骑自行车的熊/gli orsi che stanno andando in bicicletta

“the bears that are cycling”

1. 喝水的斑马/le zebre che stanno bevendo acqua

“the zebras that are drinking water”

1. 钓鱼的熊/l’orso che sta pescando

“the bear that is fishing”

1. 拿苹果的猴子/le scimmie con la mela

“the monkeys with the apple”

1. 在写字的小朋友/il bambino che sta scrivendo

“the child that is writing”

# Practice Materials

1. 拉男孩的猴子/la scimmia che sta tirando i bambini

“the monkey that is pulling the boys”

1. 女孩画的男孩/il ragazzo che le ragazze stanno pitturando

“the boy that the girls are painting”

1. 拿雨伞的小朋友/il bambino che sta tenendo l’ombrello

“the child that is holding the umbrella”
